# On the transience or stability of subthreshold psychopathology

**DOI:** 10.1038/s41598-021-02711-3

**Published:** 2021-12-02

**Authors:** Marieke J. Schreuder, Johanna T. W. Wigman, Robin N. Groen, Marieke Wichers, Catharina A. Hartman

**Affiliations:** Interdisciplinary Center for Psychopathology and Emotion regulation, Department of Psychiatry, University Medical Center Groningen, University of Groningen, Hanzeplein, 19713 GZ Groningen, The Netherlands

**Keywords:** Psychiatric disorders, Dynamical systems, Time series

## Abstract

Symptoms of psychopathology lie on a continuum ranging from mental health to psychiatric disorders. Although much research has focused on progression along this continuum, for most individuals, subthreshold symptoms do *not* escalate into full-blown disorders. This study investigated how the stability of psychopathological symptoms (attractor strength) varies across severity levels (homebase). Data were retrieved from the TRAILS TRANS-ID study, where 122 at-risk young adults (mean age 23.6 years old, 57% males) monitored their mental states daily for a period of six months (± 183 observations per participant). We estimated each individual’s homebase and attractor strength using generalized additive mixed models. Regression analyses showed no association between homebases and attractor strengths (linear model: B = 0.02, p = 0.47, R^2^ < 0.01; polynomial model: B < 0.01, p = 0.61, R^2^ < 0.01). Sensitivity analyses where we (1) weighed estimates according to their uncertainty and (2) removed individuals with a DSM-5 diagnosis from the analyses did not change this finding. This suggests that stability is similar across severity levels, implying that subthreshold psychopathology may resemble a stable state rather than a transient intermediate between mental health and psychiatric disorder. Our study thus provides additional support for a dimensional view on psychopathology, which implies that symptoms differ in degree rather than kind.

## Introduction

Psychopathology is increasingly recognized as a dimensional phenomenon^1–5^. From such a dimensional perspective, psychiatric disorders reflect the extreme end of a severity continuum ranging from the absence of symptoms to the presence of severe symptoms. Along this continuum lie subthreshold symptoms, which fall short of the diagnostic criteria for a clinical disorder but may still cause burden and functional impairments^3,6,7^.

A dimensional view on psychopathology implies that the differences between subthreshold symptoms and their full-threshold counterparts are quantitative rather than qualitative. This is supported by studies showing that subthreshold symptoms and full-blown psychiatric disorders have a similar etiology, structure (based on symptom interrelations^8^), and treatment response (i.e., phenomenological continuity^9^). For instance, mild psychiatric traits and disorders share similar genetic risk factors, illustrated by the finding that 80% of the covariance between subthreshold symptoms and psychiatric disorders is attributable to genetic overlap^10^. Similarly, the brain regions associated with subthreshold and clinical manifestations of psychopathology are largely overlapping^11^. Environmental risk factors, such as childhood abuse and stressful life events, have also been related to both sub- and full-threshold expressions of psychopathology^6,12^ Finally, like psychiatric disorders, subthreshold symptoms are associated with distress and declined functioning^6,7,12,13^, which can improve following psychological treatment^14^. In sum, there is substantive evidence that the distinction between subthreshold symptoms and psychiatric disorders seems to be a matter of *degree*—e.g., reflected in the number of symptoms and affected individuals—rather than *kind*^15,16^.

Subthreshold symptoms are commonly considered clinically relevant not only because of the above-mentioned similarities to psychiatric disorders, but also because of their prognostic significance^1^. That is, individuals with subthreshold symptoms are two to five times more likely to develop a psychiatric disorder compared to individuals without such symptoms^1,17,18^. This implies that, for some individuals, subthreshold symptoms reflect a temporary phase between having no symptoms and having a psychiatric disorder. Yet, longitudinal cohort studies have shown that for the majority of individuals, subthreshold symptoms do *not* escalate into full-blown disorders. Specifically, the proportion of individuals with subthreshold symptoms that meet the criteria for a psychiatric disorder when assessed several years later ranges between 14 and 35% (depression^1,2,19^), 14–15% (anxiety^1,20^), 32% (bipolar disorder^21^), 25% (psychosis^22^), and 36–38% (substance abuse^1^). For other individuals, subthreshold symptoms may either remit or persist. Such persistence contradicts the common notion that subthreshold symptoms are transient. Instead, subthreshold symptoms could—at least for some individuals—reflect stable states rather than transient transitionary phenomena. This introduces the possibility of yet another qualitative similarity between subthreshold symptoms and psychiatric disorders: both might be stable phenotypes.

So far, the stability of psychopathological symptoms has mostly been investigated across very short timescales (e.g., hour-to-hour) and relatively long timescales (e.g., year-to-year). The present study aims to investigate the day-to-day stability of psychopathological symptoms across six months using a complex systems perspective^23–26^. According to this perspective, symptoms might manifest as stable states, for instance labelled as mental health, subthreshold psychopathology, or psychiatric disorder^27–29^. These stable states—commonly referred to as attractors—can be thought of as the set point to which systems tend to return again and again upon perturbations (i.e., stressful or pleasant events). In healthy individuals, for instance, events may lead to temporary dips or uplifts in mood, but eventually, a state of mental health (i.e., their attractor) is restored. Attractors result from regulatory processes, reflected in interactions between elements of the system (e.g., feedback loops between mental states^24^). In the presence of strong regulatory processes, systems are resistant to change. This translates to a strong tendency to remain in an attractor (e.g., one with low symptom severity). As regulatory processes weaken, transitions from one attractor to another become more likely. Hence, the stability of an attractor can be inferred from regulatory processes, known as attractor strength^30^. Strong attractors (or, attractors with high attractor strength) can be considered stable and persistent. Weaker attractors, in contrast, are less stable and may therefore quickly disappear. It follows that strong attractors without symptoms of psychopathology can be considered favorable, as they reflect stable mental health. Strong attractors featured by severe symptoms of psychopathology, in contrast, may be unfavorable, as they reflect persistent mental ill-health. Finally, weak attractors can be considered transient conditions that easily disappear.

**Figure 1:**
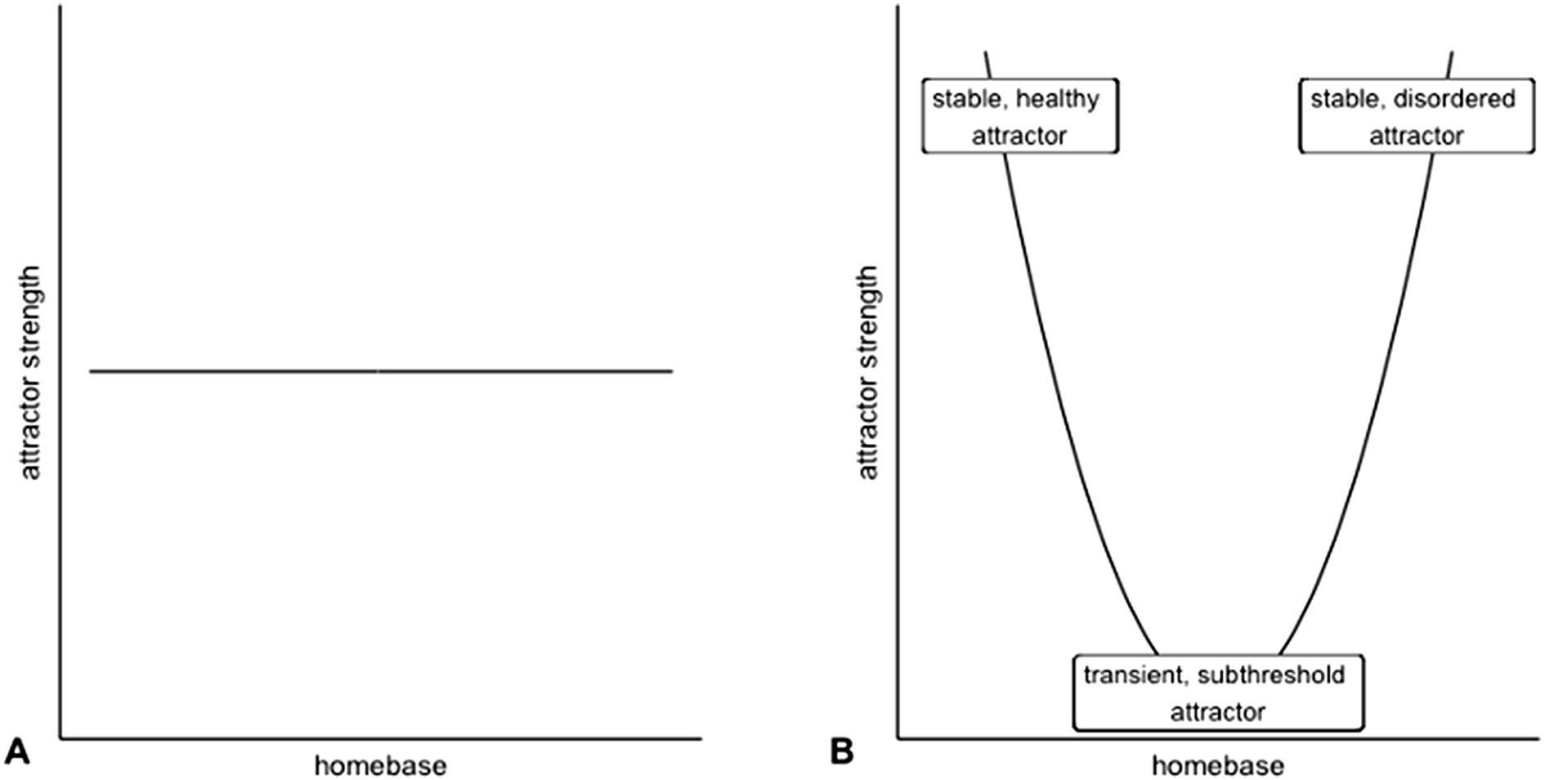
Illustration of the association between homebases and attractor strengths under two different scenarios. The homebase corresponds to the severity of symptoms that characterize an attractor. (**A**) If the subthreshold attractor is comparable to the healthy and disordered attractors in strength, there is no clear association between homebases and attractor strengths. (**B**) If the subthreshold attractor is transient, there is a quadratic relation between homebases and attractor strengths.

If subthreshold symptoms indeed reflect a stable attractor that behaves similar to the attractors with low and high symptom severity, the strengths of these attractors should be similar. This would mean that there is no clear association between the symptom severity of attractors (referred to as homebases) and attractor strengths (Fig. 1a). If, on the other hand, subthreshold symptoms reflect more transient phenomena (i.e., temporary states between low and severe symptoms), there should be a quadratic relation between homebases and attractor strengths (Fig. 1b). We investigated this hypothesis in an intensive longitudinal study where 122 at-risk young adults monitored transdiagnostic (subthreshold) symptoms daily for a period of six months. Since subthreshold symptoms are considered diffuse, representing a mix of symptoms from different psychopathological domains, we focused on attractors of overall symptom severity, rather than attractors of specific symptom domains^31^.

## Materials and methods

### Participants

Participants were recruited from the clinical cohort of an ongoing study, named TRacking Adolescents’ Individual Lives Survey (TRAILS^32^). At the time of inclusion in the clinical cohort of TRAILS (TRAILS-CC), participants were between 10 and 12 years old and had been referred to mental health care services. Because of this history, they were considered at increased risk to develop mental health problems. Since their inclusion, participants completed bi- or tri-annual follow-up assessments. When TRAILS-CC participants were approximately 23.6 years old (range 21–24), they were invited to take part in an add-on diary study (TRAILS TRANS-ID). Of the 443 eligible participants, 134 (30.2%) were included in TRAILS TRANS-ID. The present study included the 122 individuals who completed the diary period. A more elaborate description of these participants, as well as other methodological details, has been published elsewhere^33^. All participants provided written informed consent. This study was approved by the medical ethics committee of the University Medical Center Groningen (reference no. 2017/203). All procedures contributing to this work comply with the ethical standards of the relevant national and institutional committees on human experimentation and with the Helsinki Declaration of 1975, as revised in 2008.

### Procedure

Participants completed daily questionnaires every evening for a period of six consecutive months. Each questionnaire consisted of 58 items pertaining to the past day (e.g., ‘Today, I felt tired’) that were rated on a visual analogue scale (VAS) ranging from 0 to 100. These questionnaires, or diaries, were sent via a text messages to participants’ mobile phones. Prior to and immediately after the diary period (i.e., at baseline and post), a semi-structured diagnostic interview was orally administered (mini-SCAN). This interview was used to assess whether individuals met the diagnostic criteria for a DSM-5 disorder (for details on the procedure, please see^33^). The post assessment covered the entire diary period, and therefore, this assessment was used for sensitivity analyses (see “Data analysis” and Supplement).

### Data analysis

The diary procedure yielded a maximum of 183 measurements of 58 mental states per individual, for 122 individuals (i.e., > 1.2 million observations in total). The data pertaining to the 35 negative mental states assessed in our study—listed in the Supplement—were selected for analyses. Together, these mental states were considered reflective of individuals’ overall symptom severity. We estimated overall symptom severity (sx) for individual *i* at time *t* by computing the mean rating across the individual’s negative mental states (ms) at time *t*, so that sx_*i,t*_ = Σms_*i,t*_/35. Subsequently, a generalized additive mixed model (GAMM) was fitted^34,35^. Specifically, symptom severity of individual *i* at time *t* was predicted by this individual’s (1) intercept, (2) autoregressive parameter, and (3) non-linear trend in symptom severity over time (for details, see supplementSupplement). This model yielded an estimated homebase and attractor strength for each individual separately, while taking into account each individual’s change in symptoms over time. The homebase is given by the person-specific intercept (which is conceptually similar, but not equal to, the person’s mean), and reflects the symptom severity that characterizes an individual’s attractor^36^. As such, relatively low homebases can be considered adaptive, while higher homebases may be maladaptive. The attractor strength reflects the regulatory forces that maintain the attractor, and is given by person-specific estimates of the inversed autoregressive parameter (i.e., the effect of symptom severity at *t-1* on symptom severity at *t*)^36^. This operationalization of homebases and attractor strengths has also been adopted in earlier studies^36,37^, and can be considered a discrete-time translation of the parameters described in the DynAffect model^30^ and the PersDyn model^38^, which are formalized in continuous time.

The relation between homebases and attractor strengths was tested with regression analyses. Specifically, we compared models where attractor strength was predicted by homebase vs. squared homebase (i.e., polynomial regression). This allowed for differentiating between the scenarios displayed in Fig. 1. We repeated these regression analyses in two sensitivity analyses. First, we fitted weighted regressions to account for the uncertainty in the estimates and attractor strengths. The weights in these models were proportional to the sum of the range of the confidence intervals around the homebases and attractor strengths. Second, we checked the effect of (co-morbid) full-blown disorders by omitting individuals who met the criteria for at least one DSM-5 diagnosis from the analyses. This was done to allow for the possibility that mental states, and the stability thereof, might have a different meaning for individuals with versus without psychiatric disorders^39^. By re-running analyses in individuals without disorders, we could verify whether findings followed from between-individual differences in *e.g.,* the “threshold” for reporting a certain mental state. Individuals with a DSM-5 diagnosis were selected based on the mini-SCAN assessed at post, which covered the presence of psychiatric disorders during the entire diary period. All analyses were performed in R (version 4.0.2) using the package *mgcv* (version 1.8.33)*.*

## Results

Participants (N = 122, 56.6% male) were on average 23.64 years old (SD = 0.67, range = 22.26–24.81) and had on average completed 163.39 diary assessments (88.6%, SD = 17.12, range = 116–190). At baseline, 37 individuals (30.33%) met the criteria for at least one DSM-5 diagnosis. After the diary period, 34 individuals (27.87%) had a DSM-5 disorder, of whom 23 (67.65%) were also diagnosed at baseline. Most prevalent were mood disorders (n = 24 and 23 at baseline and post, respectively), followed by anxiety disorders (n = 6 and 12) and ADHD (n = 6 and 8).

The fitted values and the distribution of residuals indicated that assumptions of the GAMM were not violated (see Supplement for details). The GAMM had an adjusted R^2^ of 77% and yielded homebases that varied between 2.85 and 46.51, with a mean of 17.81 (SD = 9.80). Attractor strengths varied between 1.52 and 28.83 (mean = 4.16, SD = 3.35). Neither homebases nor attractor attractor strengths were related to the within-person variability in observations (Supplement 2, *GAMM details*). Individuals who met criteria for a DSM-5 diagnosis at post had a higher homebase (mean = 21.57) compared to non-diagnosed individuals (mean = 16.36, t(120) = 2.70, P < 0.01, Cohen’s d = 0.55), but did not differ in terms of attractor strength (mean = 4.26 vs. 4.12, respectively; t(120) = 0.21, P = 0.84, Cohen’s d = 0.04).

**Figure 2:**
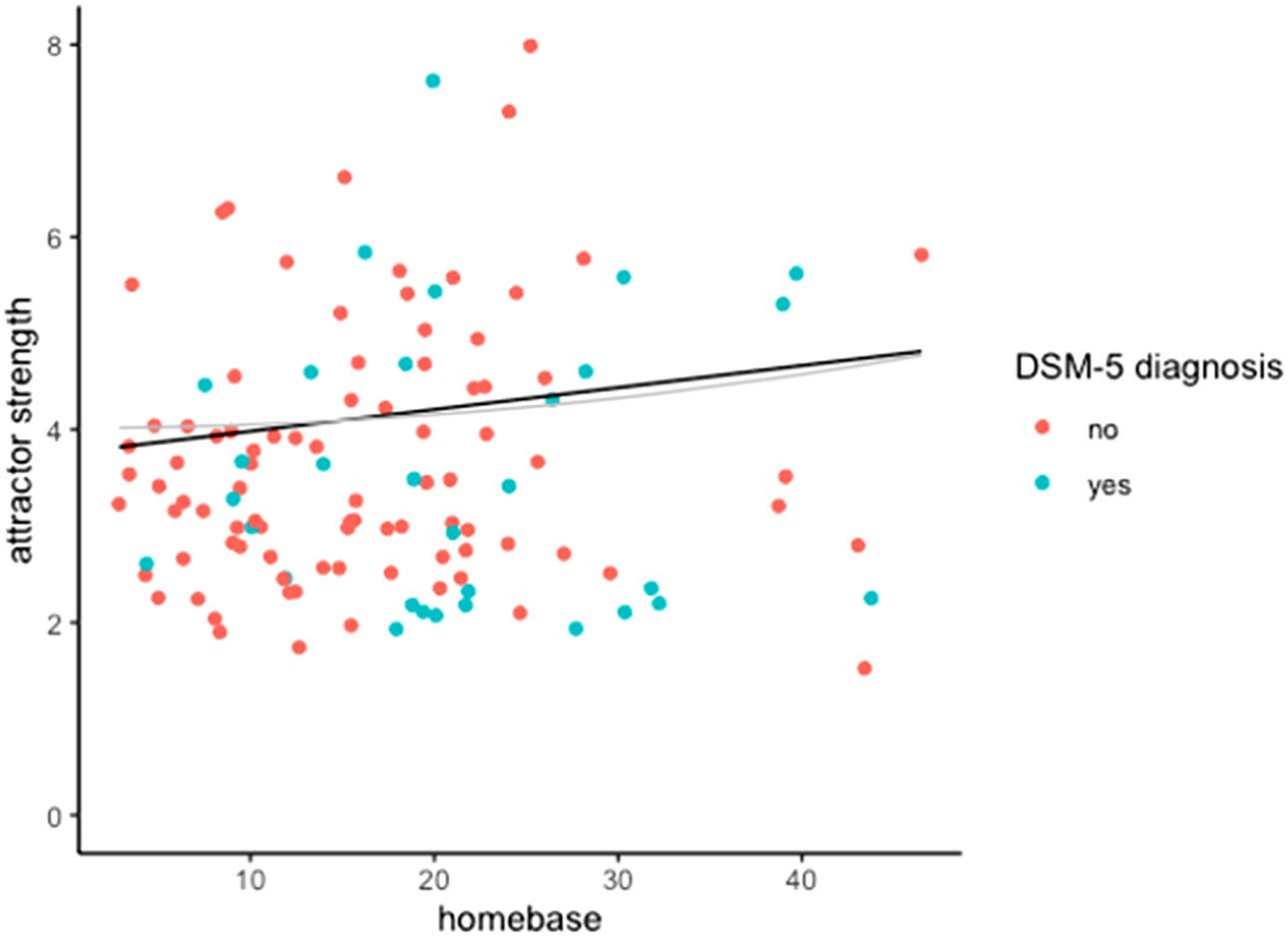
Association between the homebase and attractor strength of symptoms of psychopathology. Homebases and attractor strengths were estimated from a generalized additive mixed model using six months of daily diary data from 122 young adults. Individuals who received any DSM-5 diagnosis after the diary period are printed in blue. The black line shows the association between homebases and attractor strengths based on a linear model; the grey line shows the fit of a polynomial model. Neither model indicated an association between homebases and attractor strengths. For illustrative purposes, four outliers (individuals with an attractor strength of > 10) were omitted from this figure. Including these individuals did not change the results (see the Supplemented Figure).

Regression analyses indicated that there was no clear association between homebase and attractor strength (linear model: B = 0.02, P = 0.47, R^2^ < 0.01; polynomial model: B < 0.01, P = 0.61, R^2^ < 0.01; Fig. 2). This finding did not change after taking into account the uncertainty in the estimates nor after removing individuals with a DSM-5 diagnosis from the analyses (see Supplement).

## Discussion

Symptoms of psychopathology have been proposed to lie on a severity continuum, where the absence of symptoms and psychiatric disorders mark the extreme ends. This has been supported by the notion that subthreshold and full-threshold psychopathological symptoms show a comparable etiology^6,9–12^ and treatment outcome^14^. This study investigated the stability of psychopathological symptoms, i.e., their attraction strength, along the severity continuum. We found that the stability of symptoms assessed daily over a period of six months is independent of the severity of symptoms. This provides additional support for a dimensional view on psychopathology, which implies that subthreshold and full-threshold psychopathological symptoms differ in degree (i.e., severity) rather than in kind (e.g., stability). In conclusion, just like some individuals may experience constant mental health or psychopathology, others may get stuck in subthreshold psychopathology. Subthreshold symptoms may thus resemble stable states, rather than transient conditions that mark the progression from relatively healthy towards disordered states (or vice versa).

A dimensional view on psychopathology does not necessarily preclude the existence of discrete, stable states along the severity continuum^15,40^. Present findings show that such states not only lie on the extreme ends of the continuum—reflecting mental health and mental disorder—but may just as well occupy the regions in between these extremes—reflecting subthreshold psychopathology. It follows that the clinical relevance of subthreshold symptoms does not just lie in their associated burden^3,6,7^ and their tendency to precede full-threshold symptoms^1,17,18^ (their prognostic significance), but also in their stability. Stability here refers to a property of an attractor in a complex dynamic system, namely attractor strength. In the context of psychopathology, an attractor can be considered a set of mental states to which a system tends to return upon perturbations (e.g., pleasant/stressful events^23,27,29^). An attractor has a certain *homebase,* which may describe mild vs. more severe psychopathological symptoms, and *strength*, which reflects the regulatory processes that maintain the attractor. Relatively strong (stable) attractors with low homebase can be considered adaptive, as they illustrate a healthy system that is resilient to external perturbations. In contrast, attractors with higher symptom severity may be maladaptive, illustrated by the current finding that individuals with a DSM-5 disorder had higher homebases than those without a disorder.

We have shown that maladaptive attractors do not differ from more adaptive attractors in terms of strength. Yet, previous work has reported that individuals with a psychiatric disorder may have weaker attractors compared to healthy controls, implying a negative association between attractor strengths and homebases^36,37^. Similarly, studies that used alternative measures of stability (i.e., adjusted square of successive differences^41–43^ and probability of acute change^42,43^) found higher instability in patients compared to controls. However, this difference was likely driven by the standard deviation, meaning that patients and controls may differ primarily in the dispersion of mental states as opposed to the stability of mental states^41,44^. An explanation for the discrepancy between earlier and present findings could be that the at-risk youth in the present study are more impaired than the healthy controls and/or less impaired than the patient samples in former studies^36,37^, which in turn may have restricted the ranges in attractor strengths and homebases in the current study. However, the large variability in symptom severity in the present sample suggests that a restricted range of psychopathological symptom severity is unlikely to underlie current findings. Instead, the differences across studies concerning attractor strengths potentially follow from considerable differences in sampling frequency (i.e., assessments with a 1-day interval vs. 15-min/1-h interval) and duration (i.e., 6 months vs. one to two days): while individuals with psychiatric disorders may have a lower hour-to-hour stability of emotions compared to non-affected individuals^36,37^, their day-to-day stability of mental states may be, as indicated by the current findings, similar. Hence, while stability on a short timescale could be adaptive—for instance because it signals adequate emotional responsivity to environmental cues^45^—the meaning of stability on a longer timescale depends on the homebase that is maintained. Specifically, stability on a longer timescale can be either adaptive (when it maintains mental health) or maladaptive (when it maintains sub- or full-threshold psychopathological symptoms). In conclusion, the meaning of psychological dynamics—such as the stability of mental states—crucially depends on the timescale under consideration. An important goal for further research is therefore to investigate the timescale at which dynamics such as attractor strengths are informative of psychopathology.

Besides the timescale of assessments, the dynamics of psychopathological symptoms might be affected by the type of symptoms under consideration. It could for instance be hypothesized that certain symptom domains (e.g., anxiety) are more stable than others (*e.g.,* psychosis, mania^46^). At present, little is known about such between-domain differences: it has been reported that panic disorder and major depression show higher homebases (but similar attractor strengths) compared to borderline, post-traumatic stress and eating disorders^37,47^, while negative psychotic symptoms may have a stronger attraction (but similar homebase) compared to positive symptoms of psychosis^48^. However, small sample sizes and methodological heterogeneity preclude firm conclusions. To investigate dissociations between homebases and attractor strengths across clinical stages and psychopathological domains, future studies should aim to include individuals with a wide range of symptoms of varying severity. The current study did so by including youth who experienced a widely varying degree of (mental health) problems and a wide variety of mental states.

It should be noted that although we collected intensive longitudinal data—which allows for addressing *within*-individual processes, including changes in homebases or attractor strengths over time within individuals—we investigated differences in homebases and attractor strengths *between* individuals. Our approach fits the notion that the boundaries between mental health, subthreshold psychopathology, and full-threshold psychopathology are based on differences *between* rather than *within* individuals. This can be illustrated as follows: if an individual consistently experiences more mental health problems than others (i.e., between-person difference), without ever deviating from their own homebase (i.e., without within-person differences), they can still meet the criteria for a mental disorder. Conversely, another individual who substantially differs from their own homebase (i.e., within-person difference), but not from mentally healthy individuals (i.e., without between-person differences), will not qualify for a mental disorder. Hence, it makes sense to study subthreshold psychopathology at a between-individual level, while adjusting for within-person fluctuations in symptoms over time. Nevertheless, it would be interesting to extend the current work by investigating the within-person association between the severity and stability of psychopathological symptoms. A second consideration is that, unlike the majority of earlier studies on subthreshold symptoms, the present study considered attractors on a continuum of symptom severity, and did not classify individuals into subgroups based on pre-set cut-offs. This is particularly advantageous given the considerable heterogeneity in definitions of “subthreshold” psychopathology that plagues research on this topic^7,13,49^. Arguably, our decision to not categorize came at the cost of an unclear clinical significance of the homebase estimates, which were based on daily ratings of negative mental states. However, the fact that individuals with a DSM-5 diagnosis had significantly higher homebases than those without a diagnosis supports our inferences. Another potential limitation of the current study is that the aggregation of symptoms into global psychopathology might have obscured domain-specific associations between the homebase and strength of attractors. However, our operationalization was in line with the notion that subthreshold psychopathology may not be domain-specific^31^, and therefore, fitted with our aim to study the dynamics of symptoms of varying severity. Finally, our estimates of attractor strengths require that the timescale of assessments (daily) matches the timescale of the process of interest (i.e., strength of attraction, or the speed with which a homebase is restored). This issue is not specific to the current study, but rather applicable to all intensive longitudinal studies: within-person dynamics (including homebases and attractor strengths) can only be estimated with sufficient sampling frequency^50^. Although the present timescale (daily) is in line with our interest in long-term stability of symptoms—as opposed to momentary fluctuations in emotions^41,44^—further work on the role of timescales in studies on symptom dynamics is hopefully awaited (for a recent example, see Sperry and Kwapil^42^).

The lack of an association between homebases and attractor strengths found in the present study implies that individuals can get stuck anywhere on the severity continuum. Attractors do not, however, eternally persist: they may change over time, and such changes may involve a shift from subthreshold to full-threshold psychopathological symptoms or vice versa. Future research is needed to establish what triggers such shifts. After a shift towards a maladaptive attractor (one with a high homebase) has occurred, it is imperative to understand what maintains the attractor. A complex systems perspective on psychopathology implies that attractors emerge from interactions between mental states—meaning that individuals with stronger attractors would be expected to show greater connectivity between mental states^24^. An alternative avenue for further research concerns the comparison of attractors of different domains of psychopathology, which could expose how specific domains progress and persist, and may inform treatment.

## Supplementary Information

Supplementary Information.
