# Supplementary material for: On the transience or stability of subthreshold psychopathology
Source: Sci Rep. 2021 Dec 2;11:23306. doi: 10.1038/s41598-021-02711-3 (PMC8640053; doi:10.1038/s41598-021-02711-3)
Supplement: Supplementary file 1 — Supplementary Information. [file 41598_2021_2711_MOESM1_ESM.docx]

***Supplement***

***On the transience or stability of subthreshold psychopathology***

Marieke J. Schreuder, Johanna T.W. Wigman, Robin N. Groen, Marieke Wichers, Catharina A. Hartman

1. List of mental states

We used the data of 35 mental states, listed below.

1. I felt lonely
2. I felt empty
3. I felt down
4. I felt guilty
5. I could not bring myself to do anything
6. I felt tired
7. I avoided things
8. I was afraid of making mistakes
9. I had difficulty making decisions
10. I worried
11. My thoughts would not leave me alone
12. I was easily distracted
13. I felt restless
14. I felt nervous
15. I was easily startled
16. I felt stressed
17. I felt overburdened
18. I was easily upset
19. I felt overstimulated
20. It bothered me that things did not go as expected
21. I was bored quickly
22. I felt impatient
23. I was unable to sit still
24. I was impulsive
25. I felt irritated
26. Others felt annoyed by me
27. I had a fight
28. I felt rebellious
29. Things did not bother me
30. I had moodswings
31. I felt strange
32. I felt suspicious
33. I experienced physical pain
34. I experienced physical discomfort
35. I’m dreading something that will happen soon
36. GAMM details

The GAMM allows for estimating smooth non-linear relationships that may differ between individuals [57–59]. This non-linearity is important to model because, in data that span six months, non-linear trends in symptom severity may be common. GAMMs estimate such non-linear relations using smooths, which comprise a set of basis functions (in this case, thin-plate regression splines [38]). The wiggliness of each smooth term is determined by the number of basis functions, which was set to 10, and a penalization parameter, which was selected by generalized cross-validation. To avoid overfitting, we let the model use the same penalization parameter for all individuals. Hence, we assumed equal wiggliness (*i.e.,* an equal amount of non-linearity) across individuals. Note that this does not pose restrictions on the shape of parameters, such as an individual’s trend in symptoms over time [57].

We fitted a model where symptom severity at time *t* was predicted by (1) an intercept, (2) person-mean centered symptom severity at *t-1*, and (3) a smooth function of centered time. Between-individual variation in these three parameters was estimated through including random effects (1 and 2) and through a factor-smooth interaction (3). The latter allowed for estimating non-linear trends in symptoms over time that differed between individuals [57]. GAMM assumes that parameters do not change in a stepwise fashion, which was checked visually using plots of the fitted and observed values. Other assumptions of the GAMM, *i.e.* that residuals are normally distributed with a mean of 0, that residuals are homoscedastic across levels of the linear predictor, and that the number of basis functions is sufficient, were checked visually using residuals diagnostics plots and a diagnostic test provided in the *mgcv* package [58] (see below: assumptions of GAMM).

Homebase and attractor strength estimation

In accordance with the DynAffect model [33], homebases were operationalized as the person-specific intercept (random effect 1 above) and attractor strengths were given by the inverse of the person-specific autoregressive coefficient (random effect 2 above). Both were independent of individuals’ trend in symptoms over time (effect 3 above). Confidence intervals around the homebase and attractor strength were computed by bootstrapping. The association between homebases and attractor strengths was examined based on regression models. Post-hoc, we checked whether homebases and attractor strengths were related to the within-person variability in daily ratings. This was not the case (homebase: r = -0.002, P = .98; attractor strength: r = 0.075, P = .41).

1. Assumptions of GAMM

Figure S1 was obtained using the *gam.check* function from *mgcv.* The left plots (S1A, S1C) show that the deviance residuals were approximately normally distributed. The departure from the straight line in the upper left plot likely results from the fact that symptom assessments were bounded between 0 and 100. Plot S1B suggests that the assumption of homoscedastic residuals was not violated. Finally, we checked the whether the number of basis functions (10) was too low using the *k.check* function. The output showed edf-values ranging between 2 and 4 (all P-values > .05), and hence, the number of basis functions was considered sufficient.


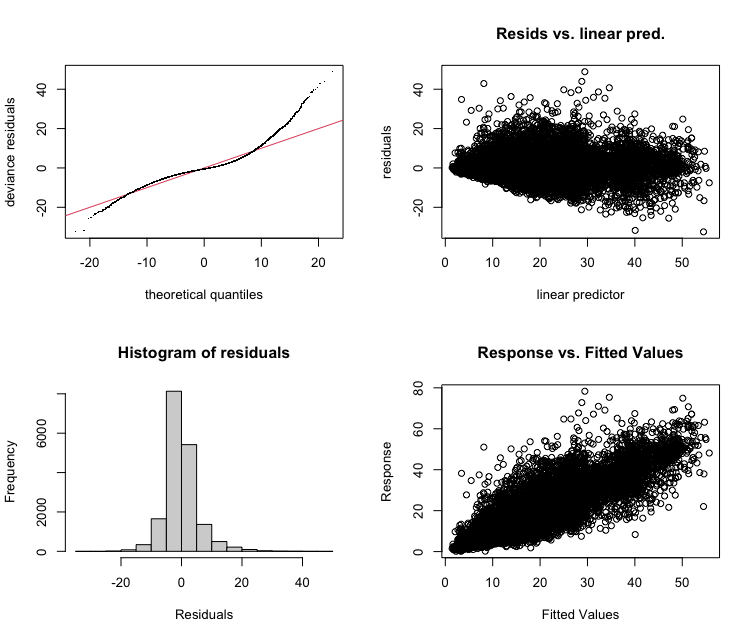


1. B.

C. D.

Fig. S1. Diagnostic information about the fit of the generalized additive mixed model. A) QQ-plot of deviane residuals. B) Residuals against the linear predictor, to evaluate the assumption of homoscedastic residuals. C) Histogram of residuals, to evaluate the assumption of normally distributed residuals. D) Outcome against fitted values.

1. Fitted values

Fig. S2. These plots show the observed values (grey dots) and fitted values (blue lines). Each plot corresponds to a single individual, resulting in 122 plots. For each plot, the x-axis depicts time (6 months, ranging from observation 1 to observation 183) and the y-axis denotes symptom severity (the outcome of the generalized additive mixed models we fitted). We denoted each individual’s estimated homebase (hb) and attractor strength (as) in the upper right corner of each plot.

1. Figure 2 including all individuals

Figure 2 in the main manuscript excluded four individuals whose attractor strength exceeded 10 (corresponding to an autoregressive coefficient of 0.10). The below figure (S2) does include these individuals. Note that the outlier estimates were very uncertain, with 95% confidence intervals ranging between -239.23 and 352.35. The mean absolute range of the 95% confidence intervals was 336.02 in the four individuals with an attractor strength > 10, and 10.27 in all other individuals (N=118). Given this uncertainty, we considered it justified to remove the estimates from the figure in the main manuscript.


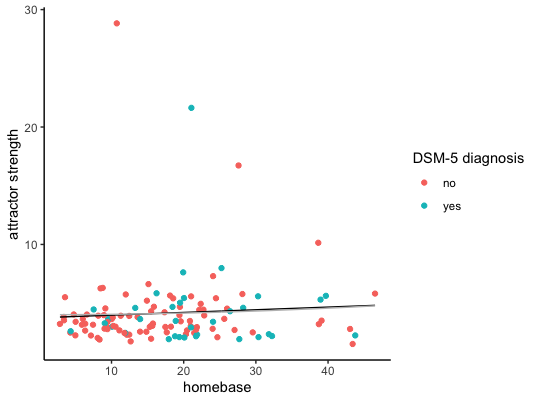


Fig. S2. Association between the homebase and attractor strength of symptoms of psychopathology. Here, we included all individuals (*i.e.* also outliers).

1. Sensitivity analyses 1: uncertainty of homebase and attractor strength estimates

Homebases and attractor strengths were estimated with varying uncertainty, which was quantified by 95% confidence intervals obtained through bootstrapping. We used the inverse of the span of these confidence intervals to compute weights. These weights were then added to the regression analyses. Similar to our main analyses, the results did not indicate an association between homebases and attractor strengths (linear model: B<0.01, P=0.69, R^2^<0.01; polynomial model: B<0.01, P=0.50, R^2^<0.01; see figure S3 below).


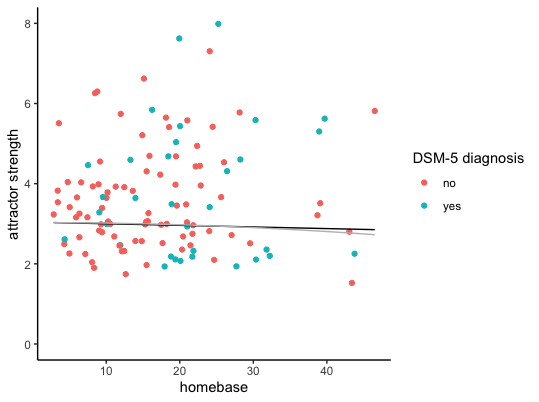


Fig. S3. Association between the homebase and attractor strength of symptoms of psychopathology. Here, we weighed observations according to their uncertainty.

1. Sensitivity analyses 2: DSM-5 diagnoses

To examine whether our results were affected by the inclusion of individuals with psychiatric disorders, we re-ran the simple and polynomial regression analyses with attractor strength as outcome and (squared) homebase as predictor and excluded 34 individuals who met criteria for a DSM-5 diagnosis during the diary period from the analyses. This resulted in a subsample of 88 non-diagnosed individuals. Similar to our findings, the regression models indicated no association between homebases and attractor strengths (linear model: B=0.03, P=0.46, R^2^=0.01; polynomial model: B<0.01, P=0.53, R^2^<0.01; see figure S4 below).


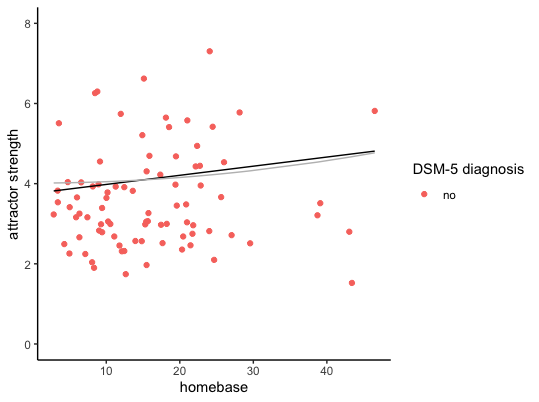


Fig. S4. Association between the homebase and attractor strength of symptoms of psychopathology. Here, we only included individuals who did not meet diagnostic criteria.
